# Dual metabolic reprogramming by ONC201/TIC10 and 2-Deoxyglucose induces energy depletion and synergistic anti-cancer activity in glioblastoma

**DOI:** 10.1038/s41416-020-0759-0

**Published:** 2020-03-02

**Authors:** Maximilian Pruss, Annika Dwucet, Mine Tanriover, Michal Hlavac, Richard Eric Kast, Klaus-Michael Debatin, Christian Rainer Wirtz, Marc-Eric Halatsch, Markus David Siegelin, Mike-Andrew Westhoff, Georg Karpel-Massler

**Affiliations:** 1 Department of Neurological Surgery, Ulm University Medical Center, Ulm, Germany; 2 IIAIG, Study Center, Burlington, VT USA; 3 Department of Pediatric and Adolescent Medicine, Ulm University Medical Center, Ulm, Germany; 4 Department of Pathology and Cell Biology, Columbia University Medical Center, New York, NY USA

**Keywords:** Cancer, CNS cancer

## Abstract

**Background:**

Dysregulation of the metabolome is a hallmark of primary brain malignancies. In this work we examined whether metabolic reprogramming through a multi-targeting approach causes enhanced anti-cancer activity in glioblastoma.

**Methods:**

Preclinical testing of a combined treatment with ONC201/TIC10 and 2-Deoxyglucose was performed in established and primary-cultured glioblastoma cells. Extracellular flux analysis was used to determine real-time effects on OXPHOS and glycolysis. Respiratory chain complexes were analysed by western blotting. Biological effects on tumour formation were tested on the chorioallantoic membrane (CAM).

**Results:**

ONC201/TIC10 impairs mitochondrial respiration accompanied by an increase of glycolysis. When combined with 2-Deoxyglucose, ONC201/TIC10 induces a state of energy depletion as outlined by a significant decrease in ATP levels and a hypo-phosphorylative state. As a result, synergistic anti-proliferative and anti-migratory effects were observed among a broad panel of different glioblastoma cells. In addition, this combinatorial approach significantly impaired tumour formation on the CAM.

**Conclusion:**

Treatment with ONC201/TIC10 and 2-Deoxyglucose results in a dual metabolic reprogramming of glioblastoma cells resulting in a synergistic anti-neoplastic activity. Given, that both agents penetrate the blood–brain barrier and have been used in clinical trials with a good safety profile warrants further clinical evaluation of this therapeutic strategy.

## Background

Glioblastoma represents a highly therapy resistant primary cancer of the brain. Patients diagnosed with this devastating disease generally, have to face an overall survival of only 1–2 years after diagnosis.^1,2^ While much progress has been made to characterise and identify molecular subgroups with resulting stratification according to MGMT or IDH status and to refine treatment modalities, such as maximising resection, introducing temozolomide or alternating electric fields, the therapeutic reality remains grim. This fact is at least partly due to the vast intratumoural heterogeneity and subsequent plasticity of these tumours allowing a selection of specific clones to thrive despite the therapeutic measures taken and in response to the new microenvironmental pressure.^3^ As a consequence, new therapeutic venues need to be taken to more efficiently hold against this disease.

Due to an intense proliferative and migratory activity, one common feature of cancers such as glioblastoma is a high demand for energy and biomass.^4,5^ The great need for substrates for the major biosynthetic pathways causes a metabolic shift towards a pro-glycolytic phenotype despite the presence of oxygen as described by Otto Warburg before.^5^ In this context, targeting cancer cell metabolism allows to attack a centre node and potentially to shut down multiple core systems at the same time to cut down on mechanisms of resistance.^6^

Imipridones such as ONC201/TIC10 are a class of small molecules that were initially developed in the 1970s as anti-seizure medication. More recently, the angular form of ONC201/TIC10 was rediscovered in a drug screen searching for TRAIL-inducing compounds and found to have anti-cancer properties.^7^ On the molecular level, ONC201/TIC10 was shown to inhibit the DRD2 receptor causing downregulation of AKT and ERK signalling resulting in FoxO3a-mediated enhanced transcription of TRAIL. ONC201/TIC10 has been clinically applied in patients with recurrent glioblastoma.^8,9^ It was shown to have a favourable safety profile and is currently in clinical trials recruiting adult (NCT03295396) and paediatric (NCT03416530) patients with gliomas harbouring H3K27M mutations.

2-Deoxyglucose represents an analogue of glucose and is characterised by the substitution of hydrogen for a hydroxyl group at the second carbon atom. It acts as an inhibitor of glycolysis at the level of phosphorylation by hexokinase. The resulting metabolite, 2-Deoxyglucose-phosphate, cannot be further processed and is trapped, which causes product inhibition of hexokinase.^10,11^ It penetrates the blood–brain barrier^12^ and has been used in multiple clinical trials including glioblastoma patients.^13,14^

In this study, we show that treatment with the imipridone ONC201/TIC10 suppresses mitochondrial respiration while it upregulates the glycolytic rate of glioblastoma cells. Moreover, dual metabolic reprogramming by targeting of OXPHOS via ONC201/TIC10 and glycolysis by 2-Deoxyglucose leads to energy depletion and enhanced anti-cancer activity in glioblastoma. Therefore, pharmacological inhibition of glycolysis might prove to be a worthy adjunct to imipridones and increase their therapeutic efficacy.

## Methods

### Reagents

ONC201/TIC10 was kindly provided by Oncoceutics, Inc. (Philadelphia, PA, U.S.A.). 2-Deoxyglucose was purchased from Sigma-Aldrich (St. Louis, MO, U.S.A.). A 10 mM stock solution was prepared for ONC201/TIC10 with dimethylsulfoxide (DMSO). For 2-Deoxyglucose a 500 mM stock solution was prepared with sterile water. All stock solutions were stored at −20 °C. For all experiments, final concentrations of DMSO were below 0.1% (v/v).

### Cell cultures and growth conditions

A172 human glioblastoma cells were obtained from the American Type Culture Collection 02/2011 (Manassas, VA, U.S.A.). The U251 human glioblastoma cell line was purchased from Sigma-Aldrich 01/2017 (St. Louis, MO, U.S.A.). The identities of the glioblastoma cell lines we purchased were confirmed by the respective source of purchase. The initial stocks were expanded, frozen and stored in liquid nitrogen. Fresh aliquots were thawed every 6 weeks. ULM-GBM-PC38, ULM-GBM-PC40 and ULM-GBM-PC128 are primary-cultured human glioblastoma cells derived from tumour resections performed at our institution and were generated and characterised as previously described.^15–18^ Human fibroblasts and human mesenchymal stem cells were kindly provided by Dr. Markus Hönicka (Department of Cardiac Surgery, Ulm University Medical Center). Primary astrocyte-enriched brain cells were provided by Dr. Matthias Schneider (Department of Neurosurgery, University of Bonn Medical Center). All cells were tested regularly for Mycoplasma contamination with negative results at the last reading 04/2019. All cells were cultured as previously described.^19–21^ Patient’s or next of kin’s consent was obtained, and procedures were done in accordance with the local ethics committee.

### Cell viability assays

In order to examine cellular proliferation, 3-[4, 5-dimethylthiazol-2-yl]-2, 5-diphenyltetrazolium bromide (MTT) assays were performed as previously described.^22,23^

### Soft agar assay

Anchorage-independent growth was examined as described before.^15^ Briefly, 2 × 10^4^ cells were seeded in a top layer of 0.35% agarose and incubated for 21d at standard culture conditions. Microscopic images were taken at ×4 magnification and colonies larger than 200 µm in diameter were counted.

### CellTiter-Glo® assay

The CellTiter-Glo® (Promega, Madison, WI) luminescent assay was used according to the manufacturer’s instructions to determine ATP levels. Briefly, the assay was performed in 96-well plates. 100 μl of CellTiter-Glo® Reagent was added to each well containing 100 μl medium and cells. Cell lysis was induced by shaking for 2 min. Then cells were incubated for 10 min at RT for stabilisation of the signal prior to measuring luminescence.

### Staining for carboxyfluorescein diacetate succinimidyl ester (CFSE)

Carboxyfluorescein diacetate succinimidyl ester labelling was performed using the CellTrace^TM^ CFSE cell proliferation kit (Molecular Probes, Inc., Eugene, OR, U.S.A.) according to the manufacturer’s instructions. Briefly, cells were enzymatically detached, pelleted by centrifugation and washed once with phosphate-buffered saline (PBS) at RT prior to resuspending 2 × 10^6^ cells/ml in PBS. CFSE was added to a final concentration of 5 µM prior to incubation for 10 min at 37 °C. The reaction was quenched by adding DMEM containing 10% FBS and incubation for 10 min at RT followed by three washes with fresh DMEM. Finally, the cells were seeded in duplicate into 6-well plates at 4.5 × 10^4^ cells/well and allowed to attach overnight. After 24 h, the treatments were started and after 96 h, the cells were detached, collected, centrifuged and resuspended in 300 µl PBS at RT followed by flow-cytometric analysis. The data were analysed using the FlowJo software (version 7.6.5; Tree Star, Ashland, OR).

### Measurement of cell death and cell cycle analysis

Cell death was detected by propidium iodide (PI) staining followed by flow cytometry as described before.^24,25^ Briefly, cells were enzymatically detached and centrifuged for 5 min at 1800 rpm prior to washing twice with PBS. Then, 300 µl PI staining solution containing 0.05% trisodium citrate-dihydrate (Carl Roth, Karlsruhe, Germany), 0.05% triton-X 100 and 0.05 mg/ml PI (Sigma-Aldrich, St. Louis, MO, U.S.A.) was added and cells were incubated for 30 min at 4 °C prior to flow-cytometric analysis.

For cell cycle analysis the FlowJo V10 cell cycle tool was used.

### Real-time PCR and cDNA synthesis

RT-PCR was performed as described before^26^ using the primers as outlined in Table 1.

**Table 1:** Primer sequences used for RT-PCR.

Gene	Forward sequence	Reverse sequence
*PHGDH*	CTT ACC AGT GCC TTC TCT CCA C	GCT TAG GCA GTT CCC AGC ATT C
*PSAT1*	ACT TCC TGT CCA AGC CAG TGG A	CTG CAC CTT GTA TTC CAG GAC C
*PSPH*	GAC AGC ACG GTC ATC AGA GAA G	CGC TCT GTG AGA GCA GCT TTG A
*18* *S*	AGT CCC TGC CCT TTG TAC ACA	GAT CCG AGG GCC TCA CTA AAC

### Western blot analysis and phospho-kinase array

Specific protein expression in cell lines was determined by western blot analysis as described before^27,28^ using the following primary antibodies: Total OXPHOS human WB antibody cocktail (1:1000, #ab110411, Abcam, Cambridge, U.K.), human caspase-3 (1:1000; #9662, CST: Cell Signaling Technology, Danvers, MA, U.S.A.), human caspase-9 (1:1000; #9508 T, clone C9, CST), β-actin (1:2000, clone AC15; Sigma-Aldrich, St. Louis, MO) and secondary HRP-linked antibodies were purchased from CST (#7076 S, #7074 S).

The proteome profiler human phospho-kinase array (R&D Systems, ARY003b) was performed according to the manufacturer’s instructions. All antibodies used for immunoblotting were applied as recommended by the manufacturer. The Bio-1D software (Vilber Lourmat, Eberhardzell, Germany) was used for quantitative analysis.

### Extracellular flux analysis

1 × 10^4^ cells were seeded on XF96 V3 PS cell culture microplates (Agilent Technologies Inc., Wilmington, DE, U.S.A.). After 24 h, cells were subjected to the indicated treatments for 24 h followed by washes with XF assay medium containing 5 mM glucose (pH adjusted to 7.5). Afterwards the mito stress test kit (Agilent Technologies Inc., Wilmington, DE, U.S.A.) was used as described by the manufacturer applying serial injections of oligomycin at a final concentration of 2 µM, FCCP at a final concentration of 2 µM and rotenone/antimycin A at a final concentration of 0.5 µM. All analyses were performed on an Agilent Seahorse XFe96 analyzer.

### Cell migration/motility assays

Cell migration was examined by a transmembraneous migration (Transwell®) assay as well as by in vitro scratch assays. Cell motility was evaluated by time-lapse live cell microscopy imaging.

For the Transwell® assay, 3 × 10^4^ cells were seeded in DMEM containing 1.5% FBS onto Transwell® membranes (Corning Incorporated, Corning, NY, U.S.A.) with a pore size of 8 µm, and intrinsically migrated towards medium containing 10–20% FBS. Experiments were carried out according to the manufacturer’s recommendations. After 24 h, the upper side of the membrane was wiped and washed with phosphate-buffered saline (PBS) for three times. The cells on the bottom side of the membrane were then fixed with methanol and stained with 4’6-diamidino-2-phenylindole (DAPI) prior to mounting. The number of migrated cells was determined by counting one high-power field at ×10 magnification in triplicate for each treatment condition using the NIH ImageJ software (http://imagej.nih.gov/ij).

Scratch assays were performed as previously described.^15^ In brief, sub-confluent cell layers in 12-well plates sustained a scratch across the well carried out with a 200 µl pipet tip. Sequential microscopic images were taken at defined time points at ×10 magnification, and the area of the scratch was further analysed with the NIH ImageJ software (http://imagej.nih.gov/ij).

For time-lapse live cell microscopy imaging, 4 × 10^4^ cells/well were seeded onto 12-well plates, and microscopic images at ×10 magnification were taken with a live-imaging inverted video microscope (Zeiss Observer.Z1, Göttingen, Germany) every 30 min for a total observation time of 24 h. During this period, cells were kept at standard culture conditions (37 °C, 5% CO_2_, water-saturated atmosphere). Single-cell tracking was performed with the MtrackJ plugin^15^ (www.imagescience.org/meijering/software/mtrackj/) for the NIH ImageJ software (http://imagej.nih.gov/ij). Normalised “wind-rose” plots were generated with the chemotaxis and migration tool from Integrated BioDiagnostics (Ibidi, Martinsried, Germany, www.ibidi.com).

### Chorioallantoic membrane (CAM) assay

In order to assess potential effects of the respective compounds and their combination on tumour formation in a three-dimensional environment, we performed CAM assays as previously described.^15^ A 1:1 mixture of serum-free medium and Matrigel® (BD Biosciences, MA, U.S.A.) containing 2 × 10^6^ cells (U251) or 1 × 10^6^ cells (ULM-GBM-PC128) was seeded onto the CAM of 1-week-old fertilised chicken eggs. The experimental treatment was started after 24 h and consisted of the local application of the respective agents twice a day. The tumour and its adjacent CAM were harvested 4d after seeding and embedded in paraffin. Sections were stained with haematoxylin and eosin and analysed microscopically (Zeiss Imager.M1, Göttingen, Germany). Microphotographs were taken at ×4 magnification. The tumour area was quantified with the NIH ImageJ software (http://imagej.nih.gov/ij).

### Toxicity study in chicken embryos

Five-day old chicken embryos were treated with solvent or the combination therapy twice a day 5d per week over a period of 11d to assess organ and haematological toxicity. Blood samples were collected to determine leukocyte and erythrocyte counts (SYNLAB, Augsburg, Germany). Blood smears were prepared, and microphotographs were taken at ×20 magnification. Morphological changes of leukocytes and erythrocytes were assessed by two independent observers. Samples from organs were extracted and fixed for at least 24 h in 10% PBS-buffered formalin. Afterwards tissues were embedded in paraffin and 4 µm thick sections were cut prior to staining with haematoxylin and eosin. Microphotographs were taken at ×10 magnification. Two independent observers examined the organs for tissue damage.

### Statistical analysis

If not stated otherwise, statistical significance was assessed by Student’s *t*-test using Prism version 5.04 (GraphPad, La Jolla, CA). A *p* ≤ 0.05 was considered statistically significant. Combination indices and isobolograms were calculated using the CompuSyn software (ComboSyn, Inc., Paramus, NJ) as described before.^26^

## Results

### Treatment with ONC201/TIC10 suppresses OXPHOS in glioblastoma cells

**Fig. 1:**
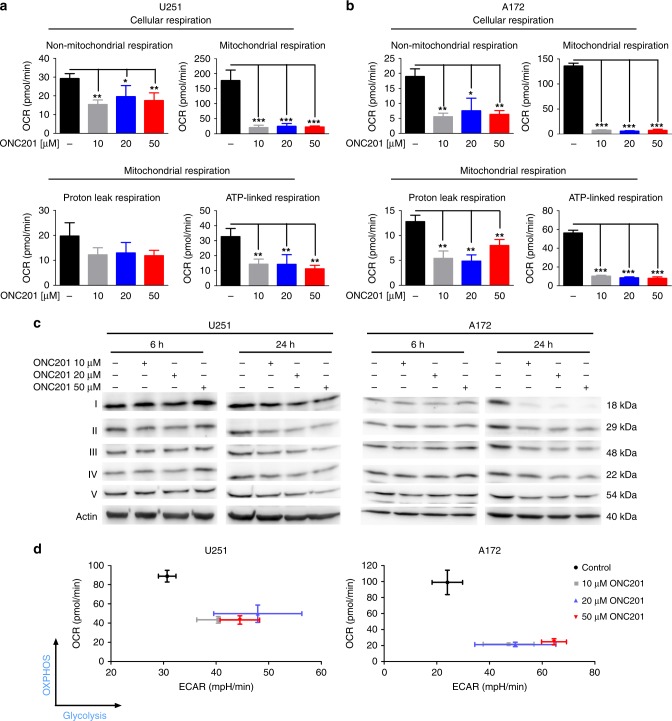
Treatment with ONC201/TIC10 reprograms glioblastoma energy metabolism suppressing OXPHOS and enhancing the glycolytic rate. **a**, **b** U251 (**a**) and A172 (**b**) glioblastoma cells were treated for 24 h as indicated with ONC201/TIC10 prior to performing mitochondrial stress tests. Oligomycin, FCCP and rotenone/antimycin A were sequentially injected accompanied by continuous extracellular flux analysis recording oxygen consumption rates (OCR). The OXPHOS inhibitors rotenone and antimycin A were used to dissect cellular respiration into non-mitochondrial and mitochondrial respiration and the ATP synthase inhibitor oligomycin was used to dissect mitochondrial respiration into proton leak and ATP-linked respiration. Columns, mean; bars, SD; *n* = 3. *p < 0.05, **p < 0.01, ***p < 0.005. **c** U251 and A172 glioblastoma cells were treated for 6 h and 24 h as indicated. Whole-cell extracts were examined by Western blot for the expression of respiratory chain complexes I–V. Actin Western blot analysis was performed to confirm equal protein loading. **d** U251 and A172 glioblastoma cells were treated for 24 h with increasing concentrations of ONC201/TIC10 followed by extracellular flux analysis measuring oxygen consumption (OCR) and extracellular acidification rates (ECAR). Graphical representation of baseline OCR/ECAR-values presented as mean and SD representative for three independent experiments.

We previously described that imipridones such as ONC201/TIC10 may affect the cellular energy metabolism of glioblastoma cells.^29^ To further dissect how the energy metabolism of glioblastoma cells is reprogrammed by ONC201/TIC10, extracellular flux analyses were performed and oxygen consumption rates (OCR) were measured. Our data show that mitochondrial respiration is significantly decreased in response to treatment with ONC201/TIC10 in U251 and A172 cells (Fig. 1a, b, Supplementary fig. 1A). Further dissection of the mitochondrial respiratory response revealed that especially ATP-linked respiration was significantly impaired when cells were subjected to treatment with ONC201/TIC10. Notably, the inhibitory effect of ONC201/TIC10 on OXPHOS did not increase in a dose-dependent manner beyond treatment with 10 µM ONC201/TIC10.

To further verify whether the inhibitory effect of ONC201/TIC10 on OXPHOS relates to the expression of specific complexes of the respiratory chain, we performed western blot analyses (Fig. 1c and Supplementary fig. 1B). After 24 h of treatment with ONC201/TIC10, expression of complex I and complex II were most strongly down-regulated in U251 and A172 cells (Supplementary fig. 1B).

### Treatment with ONC201/TIC10 upregulates the glycolytic rate of glioblastoma cells and activates the serine-one carbon-glycine (SOG) pathway

Our data showed that ONC201/TIC10 treatment led to a significant downregulation of mitochondrial respiration in U251 and A172 glioblastoma cells. To assess effects on glycolysis, extracellular acidification rates (ECAR) were measured. As shown in Fig. 1d, while OXPHOS (OCR) was significantly reduced upon treatment with ONC201/TIC10, the glycolytic rate (ECAR) was increased at basal condition.

We next addressed the question whether these ONC201/TIC10-mediated metabolic changes cause a state of bioenergetic vulnerability. To this end, we determined the cellular viability of glioblastoma cells when exposed to varying glucose concentrations. Decreasing glucose levels were associated with a marked reduction in cellular viability (Supplementary fig. 2A).

Our previous study indicated that treatment with imipridones such as ONC201/TIC10 results in a compensatory upregulation of the SOG pathway in U87 cells.^29^ To verify this finding in our setting, we performed real-time PCR analyses and determined the mRNA expression of three enzymes (*PHGDH*, *PSAT1* and *PSPH*) that are related to this pathway. As shown in Supplementary fig. 2B, treatment with ONC201/TIC10 resulted in a marked upregulation of all three enzymes in U251 and A172 glioblastoma cells.

### Combined treatment with ONC201/TIC10 and 2-Deoxyglucose synergistically reduces the viability and inhibits anchorage-independent growth of glioblastoma cells

**Fig. 2:**
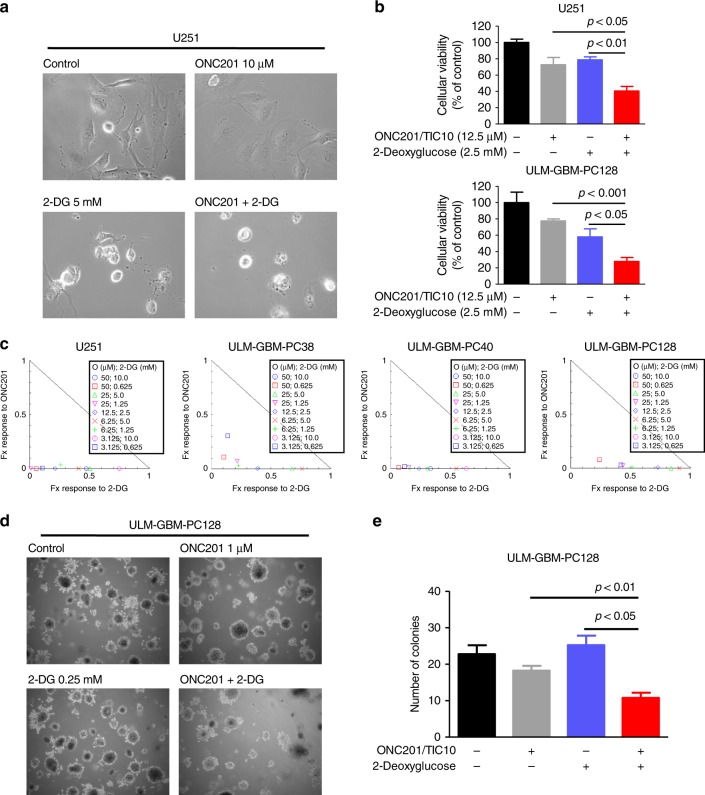
Combined treatment with ONC201/TIC10 and 2-Deoxyglucose synergistically reduces the cellular viability of glioblastoma cells. **a** Representative microphotographs of U251 glioblastoma cells treated with ONC201/TIC10, 2-Deoxyglucoe (2-DG) or the combination for 48 h at indicated concentrations. Magnification, ×40. **b** U251 and ULM-GBM-PC128 cells were examined by MTT assay after 72 h of treatment as indicated. Columns, mean; bars, SD; *n* = 4. **c** U251 and primary-cultured glioblastoma cells were treated for 72 h as indicated prior to performing MTT assays. Normalised isobolograms were calculated using the CompuSyn software. The connecting line represents additivity. Data points located below the line indicate a synergistic drug-drug interaction and data points above the line indicate an antagonistic drug-drug interaction. **d** ULM-GBM-PC128 cells were grown in agarose for 3 weeks. Representative microscopic images were taken at ×4 magnification. **e** Quantitative representation of cells treated as described for **d**. Data are representative for two independent experiments. Only colonies with diameters larger than 200 µm were counted. Columns, mean; bars, SD.

Since treatment with ONC201/TIC10 caused an upregulation of the glycolytic rate as part of a potential compensatory mechanism (Fig. 1d) and rendered glioblastoma cells vulnerable to glucose withdrawal (Supplementary fig. 2A), we hypothesised that an additional inhibition of glycolysis would induce a state of energy depletion and subsequent enhanced anti-cancer activity. 2-Deoxyglucose is a well-characterised glycolysis inhibitor that has been shown to cross the blood–brain barrier and was used in clinical trials with good tolerability.^13^ Therefore, we subjected established and primary-cultured glioblastoma cells to treatment with ONC201/TIC10, 2-Deoxyglucose and the combination (Fig. 2). Microscopic imaging revealed that cells treated with ONC201/TIC10 and 2-Deoxyglucose displayed a lower cellular density and marked morphological changes such as a pronounced rounding of the cells (Fig. 2a). This finding was reflected by a significantly reduced cellular viability of U251 and ULM-GBM-PC128 cells treated with the combination (Fig. 2b). To assess whether the combination therapy acts in a synergistic or additive manner different concentrations of ONC201/TIC10 and 2-Deoxyglucose were combined to calculate combination indices and to plot isobolograms (Fig. 2c). Combined treatment with ONC201/TIC10 and 2-Deoxyglucose resulted predominantly in a synergistic inhibitory effect on the cellular viability of the glioblastoma cells we tested (Fig. 2c and Table 2).

**Table 2:** Combination indices (CI) for U251, ULM-GBM-PC38, ULM-GBM-PC40 and ULM-GBM-PC128 glioblastoma cells treated with the indicated concentrations of ONC201/TIC10 and 2-Deoxyglucose.

ONC201/TIC10 [µM]	2-Deoxyglucose [mM]	U251	ULM-GBM-PC38	ULM-GBM-PC40	ULM-GBM-PC128
		CI	CI	CI	CI
50.0	10.0	0.47373	1.04881	0.33512	1.40290
50.0	0.625	0.05934	0.21674	0.08282	0.32259
25.0	5.0	0.50843	0.67757	0.31775	0.84413
25.0	0.125	0.01275	0.29085	0.17130	0.46780
12.5	2.5	0.21513	0.39476	0.24273	0.73843
6.25	5.0	0.41143	0.76281	0.55511	0.90978
6.25	1.25	0.29965	0.25774	0.17162	0.52630
3.125	10.0	0.74977	1.35216	0.63504	1.40078
3.125	0.625	0.11225	0.44404	0.14308	0.45498

A CI < 1 indicates synergism, a CI = 1 indicates additive action and a CI > 1 indicates antagonism.

We next addressed the question whether the combination treatment would show enhanced inhibitory activity on the colony forming ability of glioblastoma cells in soft agar. ULM-GBM-PC128 cells treated with ONC201/TIC10 and 2-Deoxyglucose showed a significantly reduced formation of colonies when compared to single-agent treatments or solvent (Fig. 2d, e).

### The combination treatment suppresses ATP levels and induces a hypo-phosphorylation signature

**Fig. 3:**
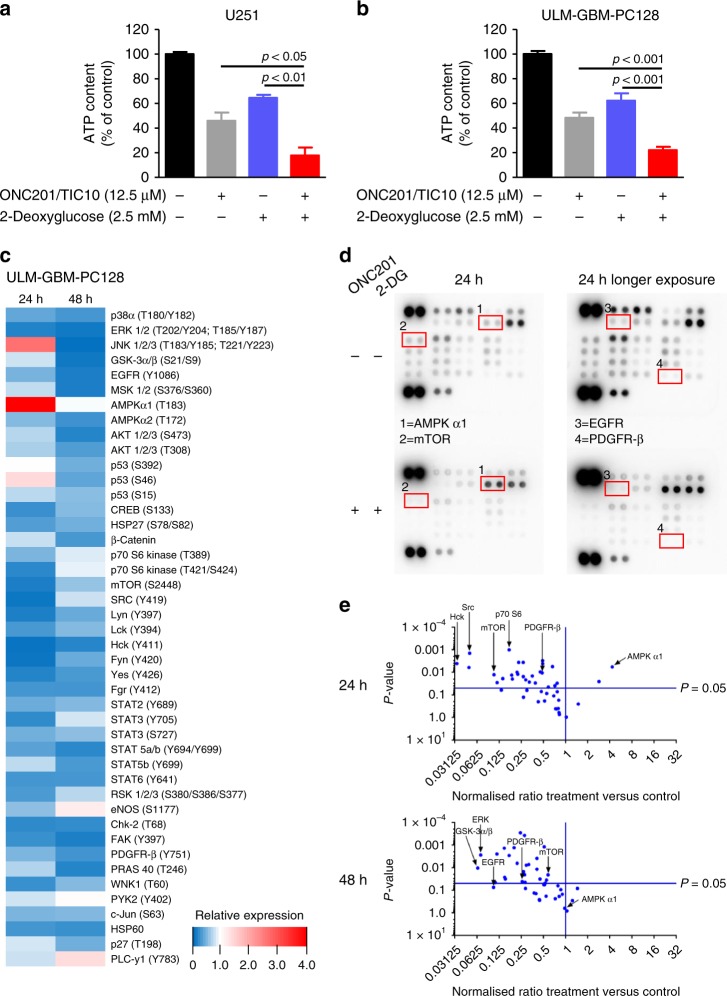
Treatment with ONC201/TIC10 and 2-Deoxyglucose causes energy deprivation and a hypo-phosphorylative state. **a**, **b** U251 (**a**) and ULM-GBM-PC128 (**b**) cells were treated for 72 h as indicated. CellTiter-Glo® assays were performed to determine ATP levels. Columns, mean; bars, SD; *n* = 3. **c**, ULM-GBM-PC128 cells were treated for 24 h or 48 h with 10 µM ONC201/TIC10 and 1.25 mM 2-Deoxyglucose combined or the respective solvent prior to collecting the lysates. After incubation of the respective lysates with the membranes of the human phospho-kinase array kit (R&D Systems, ARY003b) bound phospho-proteins were detected according to the manufacturer’s instructions. Each membrane contains kinase-specific and reference antibodies spotted in duplicate. A key to identify the coordinates of each protein spotted on the array is available on the R&D System’s website (http://www.rndsystems.com/pdf/ARY003b.pdf). Quantitative analysis using the Bio-1D software (Vilber Lourmat) was performed and the data were first normalised to the respective reference spot and second to the respective treatment control. The relative phospho-kinase expression (ON201/TIC10 plus 2-Deoxyglucose versus control) is displayed in a heatmap. **d** Representative autoradiography of the human phospho-kinase array described in **c** after 5 min/10 min exposure. **e** Volcano plots comparing Log2 fold change of phosphorylated phospho-proteins for the combination treatment versus control after 24 h and 48 h.

Next, we assessed if the combination treatment with ONC201/TIC10 and 2-Deoxyglucose leads to an enhanced inhibition of the bioenergetic state of glioblastoma cells. We therefore determined the ATP levels in U251 and ULM-GBM-PC128 cells (Fig. 3a, b). Treatment with the combination therapy resulted in a significant reduction of ATP levels when compared to cells treated with the single agents or control.

To further determine how downregulation of the energy metabolism by ONC201/TIC10 and 2-Deoxyglucose affects major protein kinase signalling pathways, we performed phospho-protein kinase arrays. Treatment with the combination of ONC201/TIC10 and 2-Deoxyglucose led to a marked increase in pAMPK α1 levels after 24 h in ULM-GBM-PC128 cells (Fig. 3c–e). Moreover, the phosphorylation status was reduced for most signalling pathways we examined including mTOR, one of the master regulators of protein synthesis, as well as major receptor tyrosine kinases such as EGFR and PDGFR-β (Fig. 3d, e). By 48 h, pAMPK levels returned back to the levels seen in control cells while the hypo-phosphorylation signature was still maintained.

### The combination treatment induces a cytostatic response

We next analysed how the combination treatment reduces cellular viability. To examine whether cell death via apoptosis or necrosis represents a relevant part of the mechanism staining for Propidium iodide and flow-cytometric analysis were performed. Neither U251 nor ULM-GBM-PC38 cells showed enhanced DNA fragmentation (Supplementary fig. 3A and B). Moreover, western blot analyses showed no significant cleavage of caspases 3 or 9 following the combination treatment (Supplementary fig. 3C). Therefore, we next assessed whether combined treatment with ONC201/TIC10 and 2-Deoxyglucose induces cytostasis. Staining with CFSE followed by flowcytometry showed that the combination treatment led to a marked increase in the fraction of cells with a higher CFSE signal, which indicates that these cells have a prolonged cell cycle progression (Supplementary fig. 3D and E). This finding was more pronounced in ULM-GBM-PC128 cells than in U251 cells.

To further examine whether this observation is associated with a cell cycle arrest, we performed cell cycle analyses. Combined treatment with ONC201/TIC10 and 2-Deoxyglucose led to an enhanced fraction of ULM-GBM-PC38 and ULM-GBM-PC128 cells in the G2/M phase after 24 h and 72 h of treatment and also after 48 h of treatment in ULM-GBM-PC128 cells (Supplementary fig. 4). However, for the most part, these changes did not exceed the ones following single-agent treatment with either ONC201/TIC10 or 2-Deoxyglucose.

### Combined treatment with ONC201/TIC10 and 2-Deoxyglucose has enhanced anti-migratory effects on glioblastoma cells

**Fig. 4:**
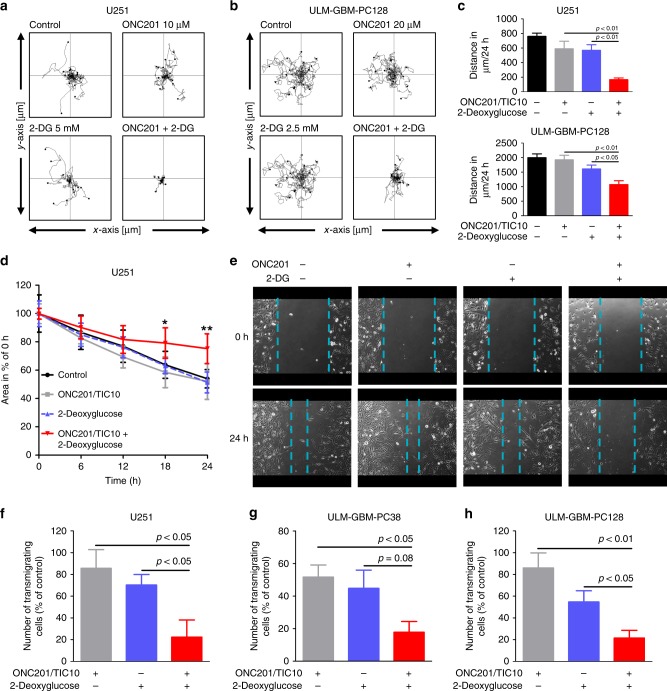
Combined treatment with ONC201/TIC10 and 2-Deoxyglucose has enhanced anti-migratory activity. **a**, **b** U251 (**a**) and ULM-GBM-PC128 (**b**) cells were seeded on 24-well plates followed by sequential microscopic imaging (magnification, ×10) over a total time period of 24 h. Single-cell tracking was performed using the MtrackJ software (see Materials and Methods). Wind-rose plots displaying the paths of 15 single cells per treatment condition during the 24 h observation period. The tracks were aligned to start from the same initial position to facilitate comparisons. **c** Total distance of 45 cells covered within 24 h per treatment condition. Columns, mean; bars, SD. **d** Monolayers of sub-confluent U251 cells were scratched prior to treatment with solvent, ONC201/TIC10 (10 µM), 2-Deoxyglucose (5 mM) or the combination. Microscopic images were taken at indicated time points after infliction of the scratch. Data are presented as mean and SD. One-way ANOVA followed by Tukey’s post hoc test was performed to assess statistical significance. *p < 0.05, **p < 0.01. **e** Representative microphotographs of cells treated as described for **d**. Magnification, ×10. **f–h** 3 × 10^4^ U251 (**f**), ULM-GBM-PC38 (**g**) or ULM-GBM-PC128 (**h**) cells were seeded onto Transwell^®^ membranes and treated with solvent ONC201/TIC10, 2-Deoxyglucose or both agents. Medium containing 10–20% FBS served as a chemoattractant. After 24 h, cells on the upper side of the membrane were wiped off, and the cells on the lower side of the membrane were stained with DAPI. Microscopic images were taken at 10x magnification and transmigrating cells were counted. Columns, mean; bars, SD; *n* = 4.

A pro-migratory phenotype is a typical characteristic of glioblastoma and contributes to the therapeutic resistance of this disease. Therefore, we tested whether the combination treatment with ONC201/TIC10 and 2-Deoxyglucose has enhanced anti-migratory activity in glioblastoma cells. To examine inhibitory effects on random movement of glioblastoma cells we performed time-lapse live cell microscopy imaging/single-cell tracking. While neither treatment with ONC201/TIC10 nor 2-Deoxyglucose significantly altered the random movement of U251 and ULM-GBM-PC128 glioblastoma cells the combination treatment strongly impaired the migration of glioblastoma cells (Fig. 4a–c).

In order to further address the question whether the combination treatment also affects directed movement, a scratch-induced migration assay was performed. U251 cells subjected to treatment with both agents showed significantly reduced migration into the scratch after 18 h and 24 h of treatment when compared to control cells and cells treated with either agent alone (Fig. 4d, e). During the whole duration of the experiment no morphological changes that may reflect beginning cytocidal effects of the combination treatment were noted.

We further examined the capacity of U251, ULM-GBM-PC38 and ULM-GBM-PC128 cells to traverse a collagen-coated porous membrane along a chemoattractant gradient. Treatment with both agents resulted in a markedly reduced transmigration of all cells tested (Fig. 4f–h and Supplementary fig. 5).

### Treatment with ONC201/TIC10 and 2-Deoxyglucose inhibits tumour growth on the chorioallantoic membrane

**Fig. 5:**
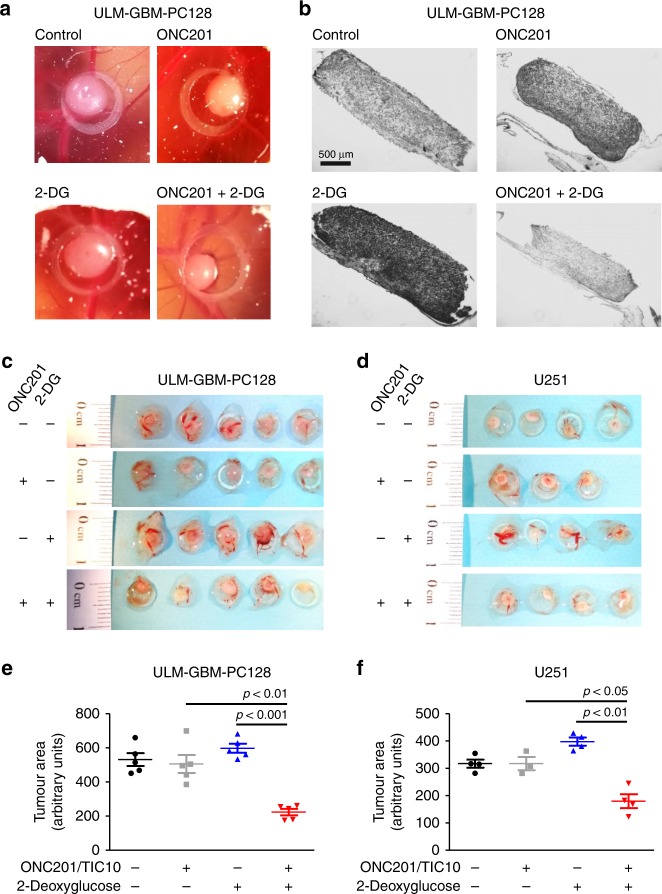
Treatment with ONC201/TIC10 and 2-Deoxyglucose inhibits tumour formation on the CAM. **a–f** 1 × 10^6^ ULM-GBM-PC128 or 2 × 10^6^ U251 cells were seeded on each CAM of fertilised chicken eggs. After 24 h, the cells were treated twice daily with 50 µM ONC201/TIC10, 25 mM 2-Deoxyglucose or both compounds. On day 5, the tumours were harvested, and photographs were taken prior to fixation in 10% formalin and histological processing. **a** Representative photographs of ULM-GBM-PC128 tumours located on the CAM at day 5. The circle has a diameter of 5 mm. **b** Representative histological images of tumours on the CAM stained with haematoxylin and eosin (magnification, ×4; scale bar = 500 µm). **c**, **d** Photographs of tumours treated as indicated. Three to five technical replicates are shown for each treatment group. **e**, **f** Quantitative representation of the tumour area as assessed by Image J (NIH, Bethesda, MD; http://imagej.nih.gov/ij). Data are presented as mean of at least three tumours per treatment group and standard deviation (SD).

The chorioallantoic membrane (CAM) assay was used to examine whether the combination treatment inhibits the tumour growth of U251 and ULM-GBM-PC128 cells in a three-dimensional “*in vivo*-near” setup. To this end, cells were seeded onto the CAM of fertilised chicken eggs and allowed to spread for 24 h prior to treatment twice daily with ONC201/TIC10, 2-Deoxyglucose, both drugs, or solvent for the following three days. As shown in Fig. 5a–f, the combination treatment led to a significantly reduced tumour size in both, the U251 and ULM-GBM-PC128 models. Moreover, the cellularity of tumours treated with the combination appeared to be reduced (Fig. 5b). In addition, the formation of blood vessels seemed to be increased following treatment with 2-Deoxyglucose alone, while an additional treatment with ONC201/TIC10 abrogated this effect (Fig. 5c, d). Notably, single-treatment with 2-Deoxyglucose led to the formation of larger tumours when compared to control toward the end of the experiment (Fig. 5e, f).

### Combined treatment with ONC201/TIC10 and 2-DG has low toxicity in normal human cells and displays no organ toxicity in chicken embryos

**Fig. 6:**
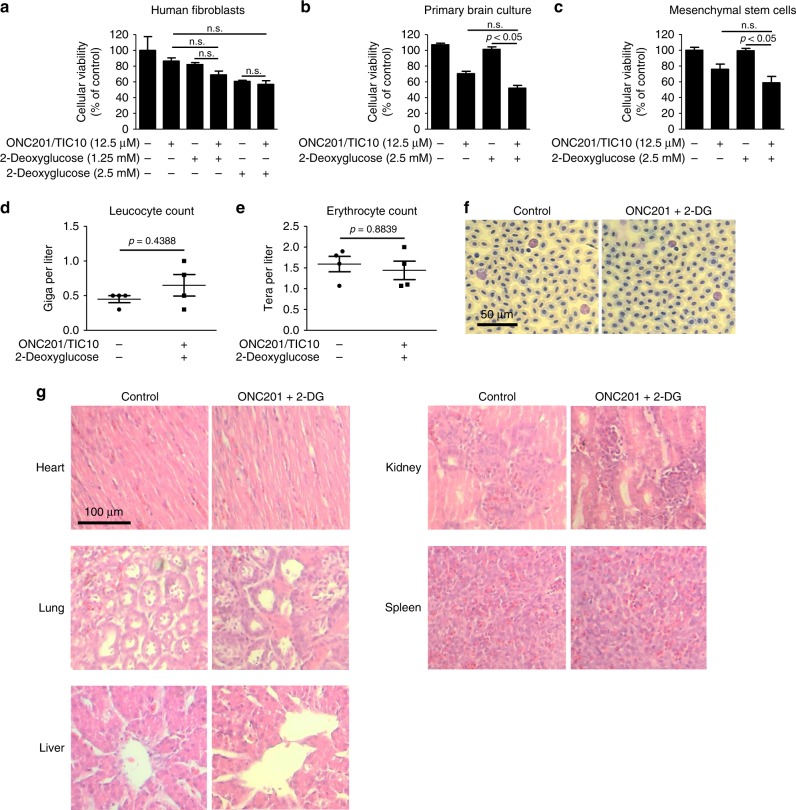
Combined treatment with ONC201/TIC10 and 2-Deoxyglucose does not display enhanced toxicity in normal human cells or chicken embryos. **a–c** Human fibroblasts (**a**), primary astrocyte-enriched brain cells (**b**) and mesenchymal stem cells (**c**) were subjected to treatment with ONC201/TIC10, 2-Deoxyglucose or the combination as indicated. After 72 h, MTT assays were performed. Columns, mean; bars, SD; *n* = 3. One-way ANOVA followed by Newman-Keuls post hoc test was used for statistical analysis. **d**, **e** Five-day-old chicken embryos were treated twice daily, 5 days per week for 11 days topically with 15 µl of 50 µM ONC201/TIC10 and 25 mM 2-Deoxyglucose. Afterwards blood samples were taken to determine leucocyte (**d**) and erythrocyte counts (**e**). Data are presented as mean and SEM. *N* = 4. **f** Representative microphotographs of blood smears derived from chicken embryos treated as described for **d**, **e**. Magnification ×40. **g** Representative microphotographs of indicated organs that were extracted from chicken embryos treated as described for **d**, **e**.

Next, we examined whether the combination treatment also affects non-pathological human cells, such as fibroblasts, primary astrocyte-enriched brain cells and mesenchymal stem cells. As shown in Fig. 6a–c, combined treatment with ONC201/TIC10 and 2-DG did affect the cellular viability of all cells tested, however, to much lower extent when compared to glioblastoma cells. Notably, the inhibitory effect of the combination treatment on the normal cells did not exceed the effects of the single-agent treatments in a significant manner.

We next addressed the question whether combined treatment with ONC201/TIC10 and 2-DG is hematotoxin or causes damage to other organs in chicken embryos. Treatment with ONC201/TIC10 and 2-DG did not significantly alter leucocyte or erythrocyte counts (Fig. 6d, e). Moreover, microscopic imaging did not reveal any difference with respect to blood cell morphology among the two treatment groups (Fig. 6f). Based on histological analyses, no damage to the heart, lung, liver, kidney or spleen was noted in organs from chicken embryos treated with ONC201/TIC10 and 2-DG (Fig. 6g).

## Discussion

In 1927, Otto Warburg first described the phenomenon of aerobic glycolysis in cancer cells.^5^ He observed that despite an abundant presence of oxygen, in cancer cells glycolysis was predominating over oxidative phosphorylation. Further analyses in more recent years touched on the complexity of the cancer cell metabolome and raised awareness for its importance as a potential cause for therapeutic resistance and as a target for cancer therapy.^4^ In terms of this study, we showed that treatment with ONC201/TIC10 resulted in a significant decrease in mitochondrial respiration, which was accompanied by a compensatory upregulation of the glycolytic rate exposing a potential metabolic vulnerability. This Achilles’ heel proved to be targetable by additional treatment with 2-Deoxyglucose resulting in a state of energy depletion and synergistic anti-cancer activity.

In the previous model systems, which we studied, treatment with imipridones resulted in concomitant downregulation of both OXPHOS and glycolysis.^29^ However, in this work we showed that treatment with ONC201/TIC10 induced an upregulation of glycolysis, which can be assumed to be part of a compensatory mechanism. These controversial results are likely to be attributable to the varying genetic background of the cellular systems used and underscore the need of using combination therapies targeting multiple metabolic pathways at the same time to efficiently impair tumour cell metabolism on a broader scale.

In this study, we show that simultaneous interference with OXPHOS and glycolysis led to a synergistic decrease of the cellular viability of established and primary-cultured glioblastoma cells. In line with our data, Kim et al. showed that bioenergetic deprivation using the anti-diabetic Metformin combined with 2-Deoxyglucose resulted in a significantly decreased cellular viability of glioblastoma tumour spheres.^30^ Similar to our results these effects were linked to significantly reduced ATP levels. Notably, treatment with Metformin and 2-Deoxyglucose resulted in impaired sphere formation and in downregulation of stemness-related genes and proteins such as CD133, Sox-2 and Notch2 to various degree. While we have not studied the effects of our therapeutic strategy on stemness it seems tempting to assume that the energy depletion effected by ONC201/TIC10 and 2-Deoxyglucose will have similar effects. This assumption is supported by the fact that ONC201/TIC10 on its own was shown to inhibit the expression of cancer stem cell-related genes/pathways and self-renewal in colorectal cancer, prostate cancer and glioblastoma cells.^31,32^ It seems probable that these effects are enhanced by an additional treatment with 2-Deoxyglucose in response to the energy depletion.

While for the most part at the concentrations used in our studies neither ONC201/TIC10 nor 2-Deoxyglucose alone displayed significant anti-migratory activity the combination of both led to remarkable anti-migratory effects. These findings are in line with others showing that targeting glycolysis or OXPHOS alone is not sufficient to inhibit the migratory capacity of glioblastoma cells but may even promote migration.^33,34^ However, similar to our data, combined inhibition of OXPHOS and glycolysis using for instance oligomycin plus 2-Deoxyglucose had synergistic effects on suppressing cell growth and motility.^33^ With respect to potential underlying mechanisms, recent work showed that 2-Deoxyglucose down-regulates miR-7-5p which in turn promotes a pro-invasive and migratory phenotype of glioblastoma cells. Whether ONC201/TIC10 when combined with 2-Deoxyglucose restores or even elevates miR-7-5p levels needs to be addressed by future studies.^34^

Targeting cancer cell metabolism seems particularly interesting in situations where the metabolic state of cancer cells is already compromised. We have shown before that glioblastoma cells carrying the IDH1 mutation display a reprogrammed cell metabolism and decreased mitochondrial respiration.^20^ Therefore, patients with IDH-mutated glioblastomas may prove to be particularly responsive to therapeutic strategies as the one presented in this study. In that context, characterisation of tumour specimens obtained from surgical resection should in future undergo metabolomic screening to unveil vulnerabilities that can be targeted in a personalised approach. Another group of patients that may benefit from such an undertaking are those with tumours displaying passenger mutations of *ENO1*.^35^ *ENO1* and *ENO2* are genes encoding the glycolytic enzyme enolase, which converts 2-phosphoglyceric acid into phosphoenolpyruvate. *ENO1* is located on 1p36 and is responsible for the vast majority of enolase activity in glioblastoma. In 1–5% of glioblastomas, the 1p36 locus was reported to be homozygously deleted which frequently includes *ENO1*. As a consequence, patients with such tumours should be more responsive to glycolysis inhibition despite the fact that *ENO2* compensates for *ENO1* deficiency to some extent. Overall, it might prove worthwhile to combine ONC201/TIC10 with enolase inhibitors, once such inhibitors will be available for clinical application and preclinical studies were performed, to treat patients with such tumour characteristics.

From a translational perspective, key advantages of our proposed therapeutic strategy are that both ONC201/TIC10 and 2-Deoxyglucose were shown to cross the blood–brain barrier and both were applied in clinical settings with good tolerability.^9,13^ For ONC201/TIC10, intratumoural drug levels in patients with recurrent glioblastoma were shown to reach up to 9.3 µM, which is close to the concentrations we used for most of our in vitro studies showing significant anti-neoplastic activity when combined with 2-Deoxyglucose.^36^ Whether the 2-Deoxyglucose concentrations used in our studies can be reached within the tumour tissue in brain tumour patients is currently not known and needs to be addressed by future clinical trials. The features mentioned before facilitate transition of this approach into a clinical trial. Of course, the profound effects of this combination therapy on the cellular metabolic circuitry may lead to unwanted side effects which we are unable to anticipate at this point. However, our preliminary toxicity studies in normal human cells and chicken embryos warrant further investigation. One limitation of this study is the fact that while we have shown that the combination therapy impairs the formation of tumours in an in vivo-near setting, we have not studied the therapeutic efficacy of our proposed strategy in an orthotopic glioblastoma model.

Overall, this study provides proof of principle that energy depletion can be achieved by a treatment with imipridones when combined with glycolysis inhibition to yield significant anti-cancer activity at multiple levels. From a mechanistic point of view, there are still open questions that remain to be addressed with respect to how the combination therapy affects OXPHOS and glycolysis. Liquid chromatography coupled with mass spectrometry would likely allow deciphering at what level the combination therapy interferes with metabolic pathways and possibly unveil additional metabolic vulnerabilities or salvage pathways, which could be targetable by an extended combinatorial therapeutic approach to further enhance the anti-neoplastic efficacy.

## Supplementary information

Supplemental Material

## Data Availability

The data supporting the results reported in the article are deposited at the translational brain tumour research laboratory, Department of Neurological Surgery, Ulm University Medical Center, Albert-Einstein-Allee 23, D-89081 Ulm, Germany.
