# Supplementary material for: Dual metabolic reprogramming by ONC201/TIC10 and 2-Deoxyglucose induces energy depletion and synergistic anti-cancer activity in glioblastoma
Source: Br J Cancer. 2020 Mar 2;122(8):1146–57. doi: 10.1038/s41416-020-0759-0 (PMC7156767; doi:10.1038/s41416-020-0759-0)

Supplementary figure legend

Supplementary figure 1: Treatment with ONC201/TIC10 suppresses oxidative phosphorylation and reduces the expression of respiratory chain complexes. A, Oxygen consumption rate (OCR) graphs of U251 and A172 glioblastoma cells that were treated for 24h as indicated with ONC201/TIC10 prior to performing mitochondrial stress tests. Oligomycin, FCCP and rotenone/antimycin A were sequentially injected accompanied by continuous extracellular flux analysis recording OCRs. Data are representative for 3 independent experiments and are displayed as mean and SD. B, U251 and A172 glioblastoma cells were treated for 24h as indicated. Whole-cell extracts were examined by Western blot for the expression of respiratory chain complexes I-V. Actin Western blot analysis was performed to confirm equal protein loading. The pixel density was quantified by Image J (NIH, Bethesda, MD; http://imagej.nih.gov/ij). Columns: mean. Bars: SD. N=3. One-way ANOVA followed by Newman-Keuls post hoc test was used for statistical analysis.

Supplementary figure 2: A reduction in glucose concentrations increases the response towards ONC201/TIC10. A, U251 and ULM-GBM-PC128 cells were treated with increasing concentrations of ONC201/TIC10 in the presence of 5mM, 1mM or 0mM glucose for 72h. Non-linear regression was performed and IC_{50}-values were calculated. Data are presented as mean and SD. N=3. B, U251 and A172 glioblatoma cells were treated for 7h, 24h and 48h with solvent or 10µM ONC201/TIC10. RNA was isolated and rtPCR was performed for *PHGDH*, *PSAT1* and *PSPH*. Colums: mean. Bars: SD. N=3.

Supplementary figure 3: Combined treatment with ONC201/TIC10 and 2-Deoxyglucose prolongs cell cycle progression. A, U251 and ULM-GBM-PC38 cells

were treated for 48h as indicated. Staining for Propidium iodide was performed prior to flowcytometric analysis. Representative histograms are shown. B, Quantitative representation of the fraction of subG1 cells in U251 and ULM-GBM-PC38 cells treated as described for A. Treatment with 50nM vincristine served as a positive control. Columns: mean. Bars: SEM. N=3. C, U251 and ULM-GBM-PC128 cells were treated as indicated. Whole cell extracts were analysed for Caspase-9 (C9), cleaved Caspase-9 (cC9), Caspase-3 (C3) and cleaved Caspase-3 (cC3). Actin served as loading control. D, U251 and ULM-GBM-PC128 cells were labeled with Carboxyfluorescein diacetate succinimidyl ester (CFSE) prior to treatment with 10µM ONC201/TIC10 and 5mM (U251) / 1.25mM (ULM-GBM-PC128) 2-Deoxyglucose or solvent. After 96h of treatment, flow cytometric analysis was performed. Representative histograms are shown. E, Quantitative representation of cells treated as described for D. Columns: mean. Bars: SD.

Supplementary figure 4: Combined treatment with ONC201/TIC10 and 2-Deoxyglucose does not induce a cell cycle arrest. ULM-GBM-PC38 and ULM-GBM-PC128 cells were treated for 24h, 48h or 72h as indicated. Cell cycle analysis was performed using the Dean-Jett-Fox model. Staining for Propidium iodide was performed prior to flowcytometric and cell cycle analysis. The data are representative for 3 independent experiments and presented as mean and SEM.

Supplementary figure 5: Combined treatment with ONC201/TIC10 and 2-Deoxyglucose inhibits directed migration. $3x10^4$ U251, ULM-GBM-PC38 or ULM-GBM-PC128 cells were seeded onto transwell® membranes and treated with solvent ONC201/TIC10, 2-Deoxyglucose or both agents. Medium containing 10-20% FBS served as a chemoattractant. After 24h, cells on the upper side of the membrane were

wiped off, and the cells on the lower side of the membrane were stained with DAPI. Representative microscopic images are shown. Magnification 10x.

Supplementary figure 1

A

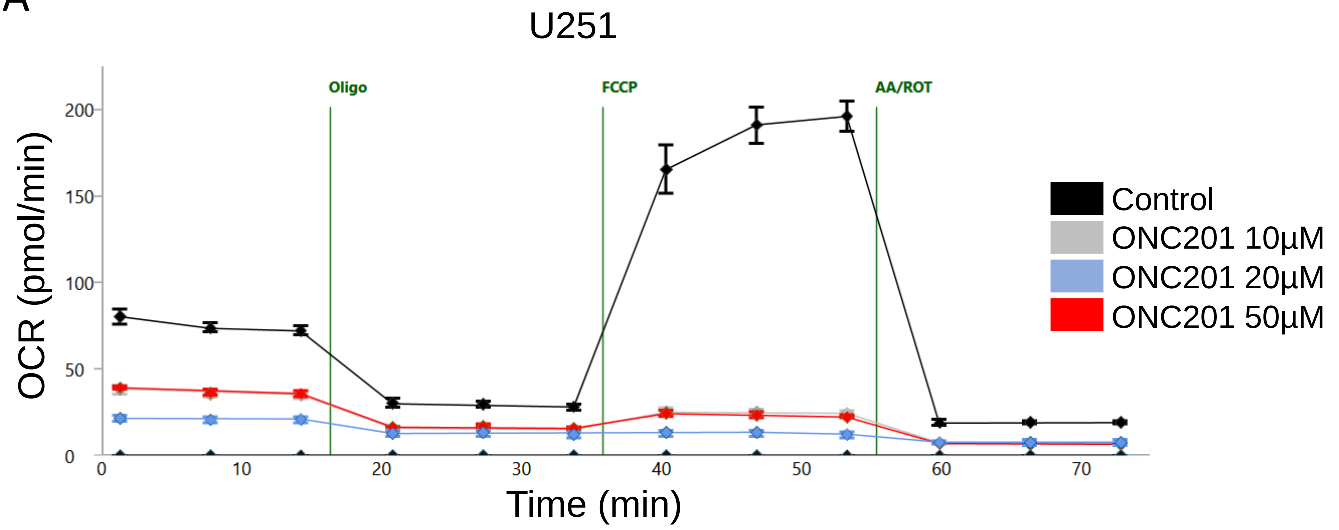

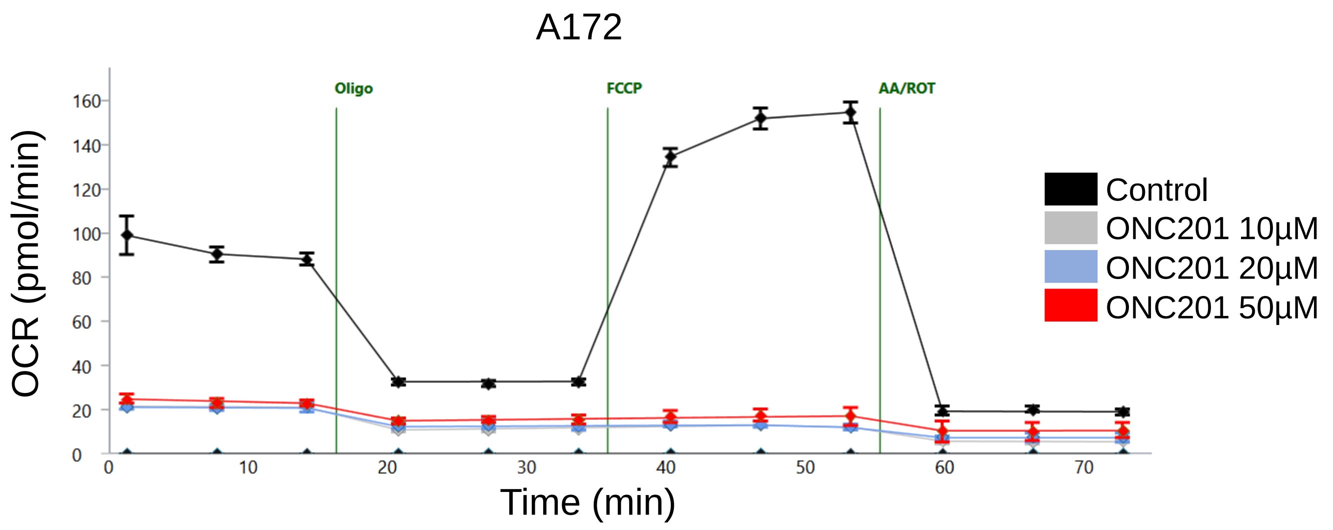

B

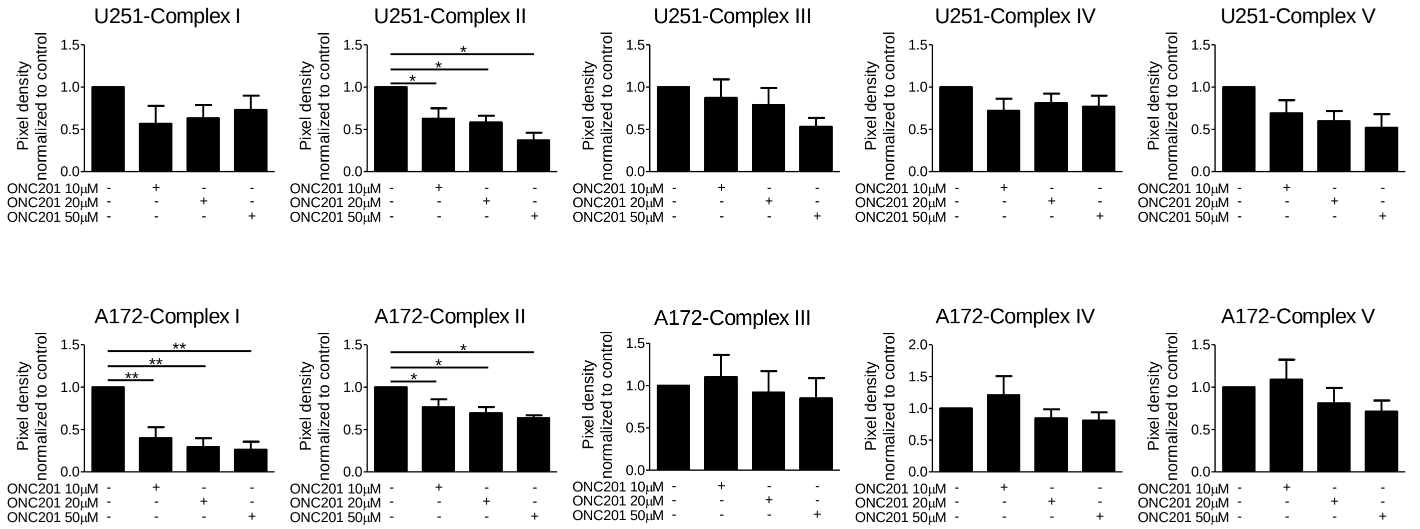

Supplementary figure 2

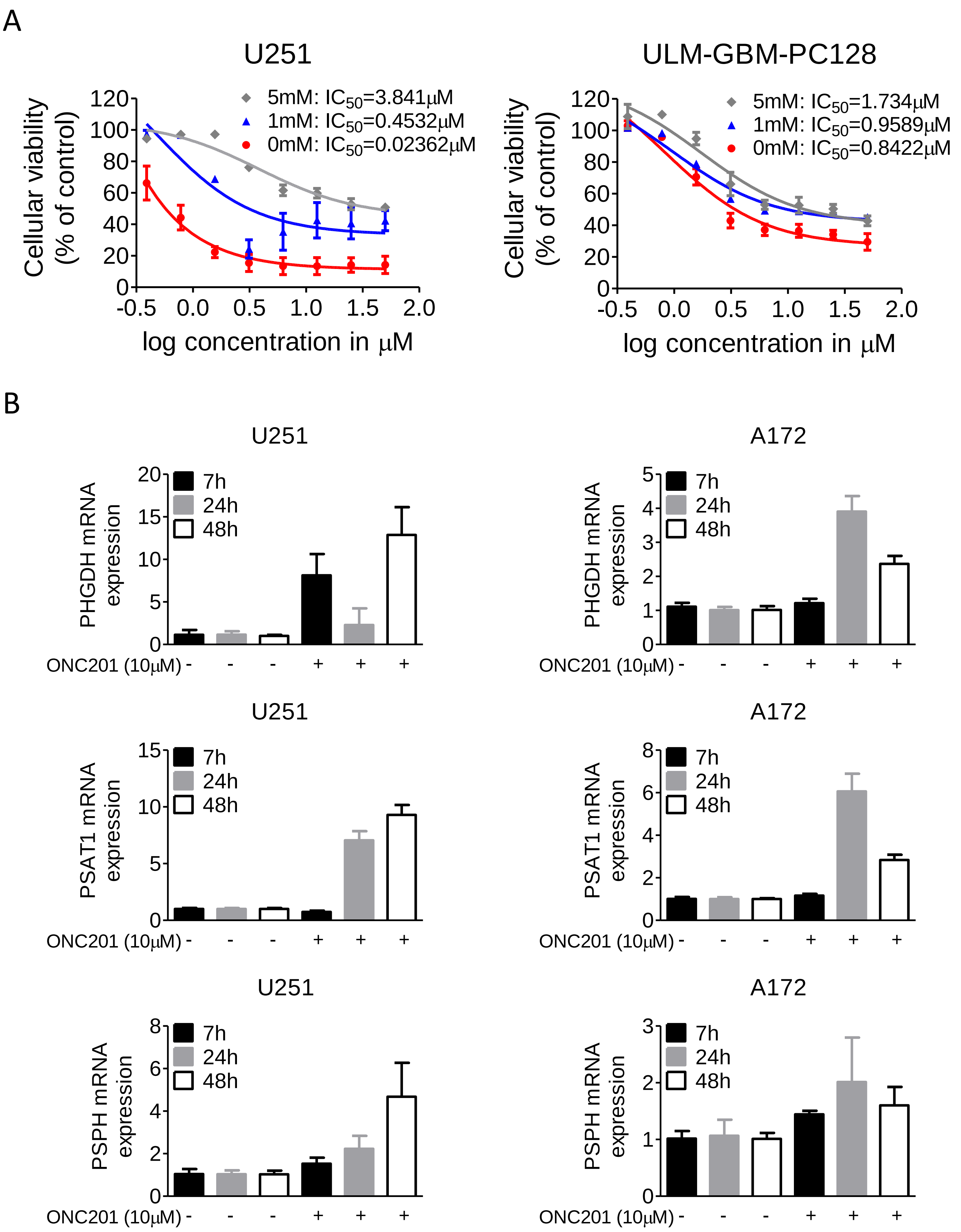

Supplementary figure 3

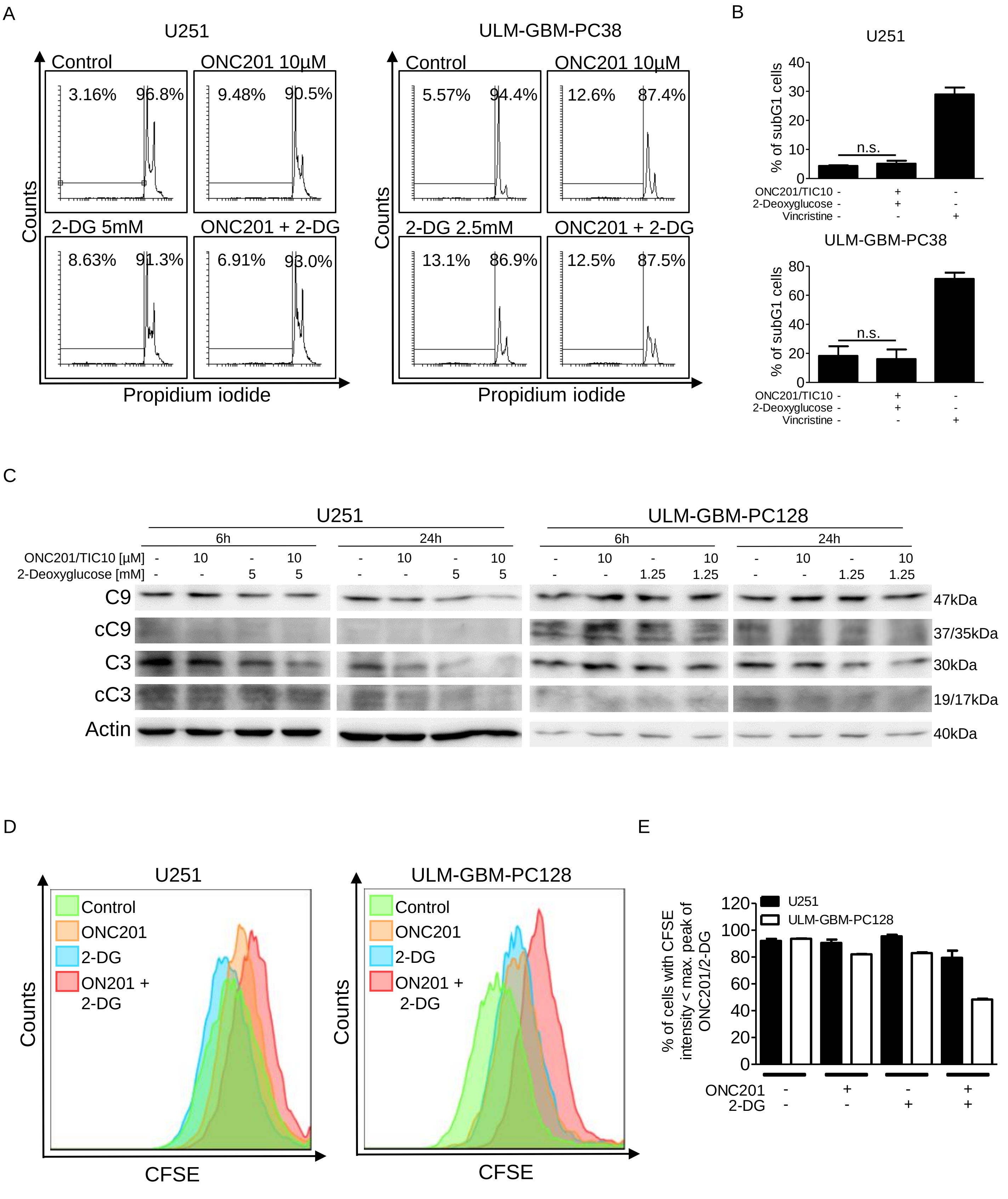

Supplementary figure 4

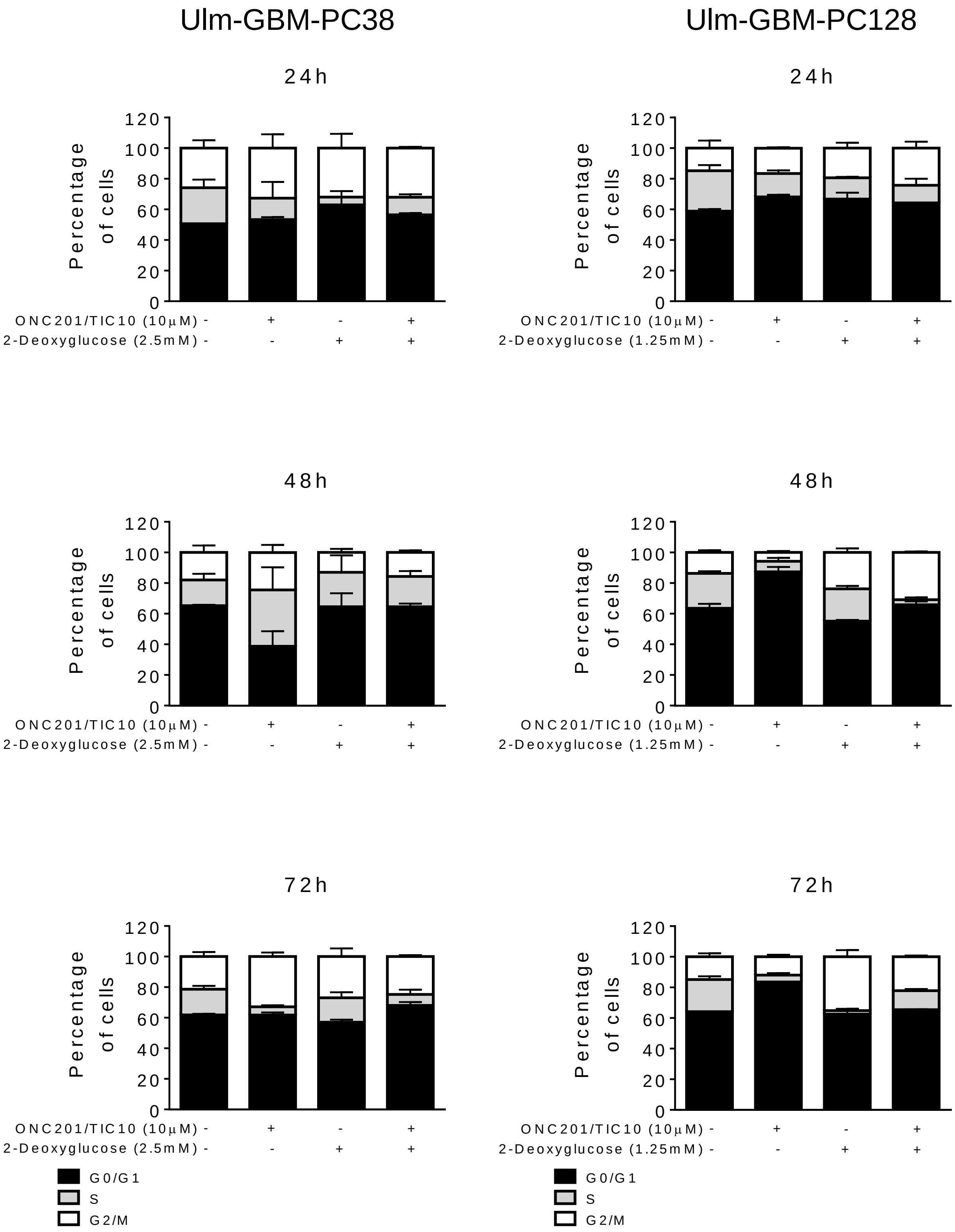

Supplementary figure 5

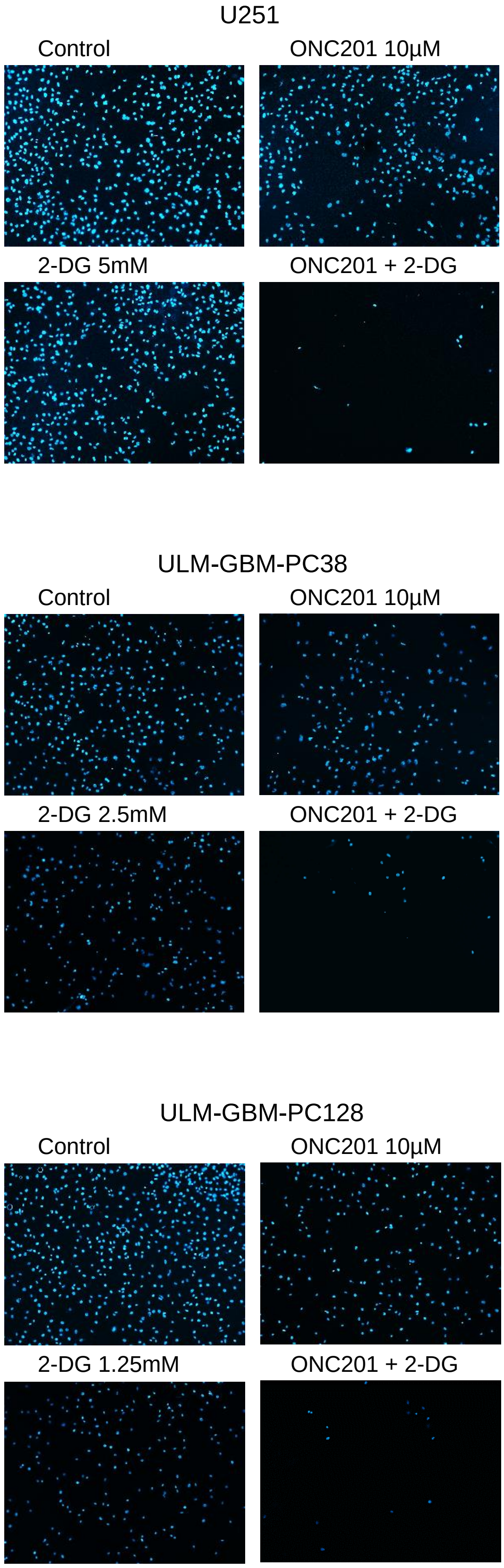

Figure 1C

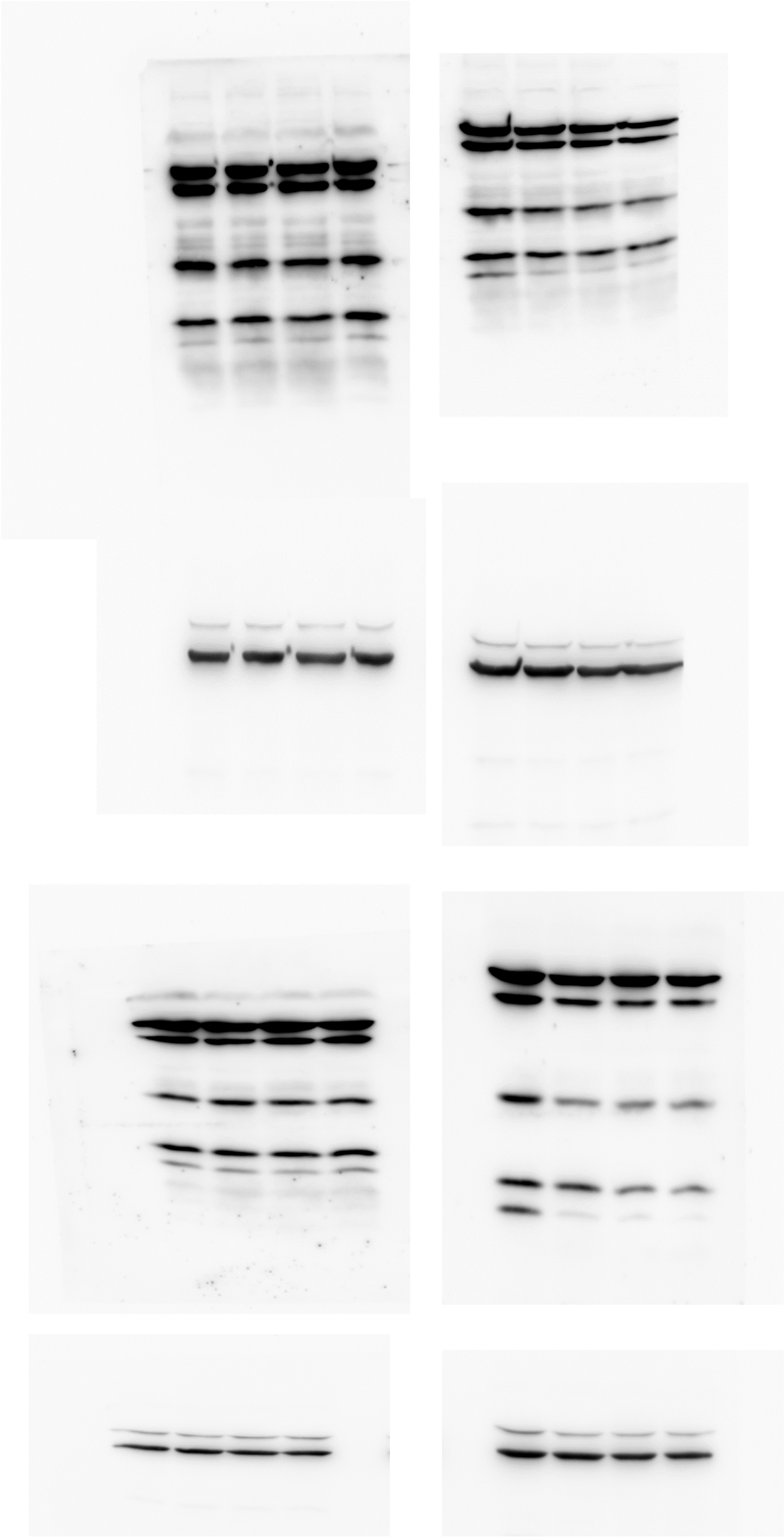

Supplement: Supplementary file 1 — Supplemental Material [file 41416_2020_759_MOESM1_ESM.pdf]
